# Self-reported cardiovascular health of teachers: results from the 5-year follow-up of the Gutenberg Health Study cohort

**DOI:** 10.1007/s00420-020-01576-9

**Published:** 2020-10-26

**Authors:** Merle Riechmann-Wolf, Sylvia Jankowiak, Andreas Schulz, Janice Hegewald, Karla Romero Starke, Falk Liebers, Karin Rossnagel, Alicia Poplawski, Natalie Arnold, Matthias Nübling, Andreas Seidler, Manfred Beutel, Norbert Pfeiffer, Karl Lackner, Thomas Münzel, Kathrin Bogner, Philipp S. Wild, Ute Latza, Stephan Letzel

**Affiliations:** 1 Institute for Teachers’ Health, University Medical Center of the Johannes Gutenberg University of Mainz, Kupferbergterrasse 17-19, 55116 Mainz, Germany; 2 Federal Institute for Occupational Safety and Health, BAuA Berlin, Berlin, Germany; 3 University Medical Center of the Johannes Gutenberg University of Mainz, Mainz, Germany; 4 IPAS Dresden: Institute and Polyclinic of Occupational and Social Medicine, Carl Gustav Carus Faculty of Medicine, TU Dresden, Dresden, Germany; 5 FFAW: The Freiburg Research Center for Occupational Sciences, Freiburg, Germany; 6 Institute of Occupational, Social, and Environmental Medicine, University Medical Center of the Johannes Gutenberg University of Mainz, Mainz, Germany

**Keywords:** Cardiovascular risk factors, Cardiovascular diseases, Teachers, Social occupations, Occupational medicine

## Abstract

**Objectives:**

Following an exploratory approach, we examined cardiovascular disease risk factors at baseline and the 5-year incidence proportion of self-reported doctor-diagnosed cardiovascular diseases (CVD) in teachers and other occupational groups of the Gutenberg Health Study.

**Methods:**

Study participants lived in the region of Mainz, Germany. Data from 6510 working participants without prevalent CVD at baseline (2007–2012) were analyzed. Participants were teachers (*n* = 215), other professionals from the health, social or educational (HSE) fields (*n* = 1061) or worked outside the HSE fields (*n* = 5234). For occupational comparisons, we estimated prevalence ratios (PR) for each CVD risk factor at baseline with robust Poisson regression analyses. We calculated crude CVD incidence rates based on the observed 5-year CVD cumulative incidence at follow-up and estimated age-weighted incidence proportions. All analyses were stratified by sex.

**Results:**

Male non-HSE workers showed a higher prevalence of smoking and physical inactivity than male teachers (PR 2.26; 95%-CI: 1.06–4.82/PR 1.89; 95%-CI: 1.24–2.87). In contrast, non-HSE workers and other HSE professionals were less likely to have reported an unhealthy alcohol intake than teachers. Differences were attenuated after SES-adjustment. We did not detect occupational group-specific differences in CVD incidence. However, there were only two cases of CVD among the teachers.

**Conclusion:**

Particularly male teachers showed a healthier lifestyle regarding physical inactivity and smoking. Nevertheless, occupational-medical care practitioners and researchers need to be aware of the relatively heightened prevalence of unhealthy alcohol intake in female and male teachers, and in absolute terms, the high hypertension prevalence in male teachers.

## Background

In Germany, teachers represent a large professional group typically in civil service. In the Western German federal states like Rhineland-Palatinate (RLP), most of the teachers (78–93%) are civil servants (statista 2020). Teachers with civil servant status are generally employed for life and are protected by special insurance in the case of illness, invalidity and retirement. Teachers’ health is assumed to be relevant for their performance and work ability (Seibt et al. 2016), and teachers’ attitude towards health-related issues may be also essential for conveying health promoting principles to pupils (Gilbert et al. 2015). Teaching itself is characterized by interactive and emotional labor that may be associated with stress (van Droogenbroeck and Spruyt 2015). Psychosocial work strain among teachers may also arise from time pressure, a lack of time control, a lack of freedom at work, from little social support by colleagues, and from noise (e.g., Scheuch et al. 2015; Nuebling et al. 2013). In addition, there is established evidence on existing associations between psychosocial work strain and cardiovascular diseases (CVD) or CVD risk factors (particularly smoking, physical inactivity, obesity and diabetes) in general (e.g., Fishta and Backé 2015; Theorell et al. 2016; Nyberg et al. 2013). Only few studies have examined aspects of cardiovascular health in teachers so far. In a study by Helmert et al. (1997) male teachers showed the lowest CVD prevalence compared to 30 other occupations, while Scheuch et al. (2015) reported CVD prevalence in teachers was similar to the general population. Furthermore, some studies suggested a lower prevalence of CVD risk factors in teachers, but these results were partly inconsistent (Gilbert et al. 2015; Seibt et al. 2005, 2016; Kovess-Masféty et al. 2006; Akintunde and Oloyede 2017; Brown et al. 2006; Scheuch et al. 2015). Occupational differences in the prevalence of CVD or CVD risk factors reported in these previous studies were partly attenuated by adjustment for SES, but not all of the study estimates failed to reach statistical significance after adjustment.

Overall, a comprehensive interpretation and transferability of these study results is difficult due to their cross-sectional study design, different socio-economic contexts, use of heterogeneous comparison groups or the results are no longer up to date. Thus, we aimed to contribute to the previous research with more recent results from the Gutenberg Health Study (GHS) cohort. We compared teachers with the general working GHS population to estimate the CVD-related effect of working as a teacher. We also compared teachers with other professionals working in the *h*ealth, *s*ocial or *e*ducational fields (HSE), as occupational circumstances may be similar for social occupations in general (van Droogenbroeck and Spruyt 2015). Any possible differences between teachers and other HSE professionals might indicate if factors unique to teaching or the teachers themselves have an effect on cardiovascular health. Finally, we examined and contrasted the occupational group-specific prevalence of CVD risk factors at baseline as well as the 5-year incidence proportion of self-reported doctor-diagnosed CVD at follow-up. If our results indicate any heightened risk for teachers they should be helpful to define potential approaches for preventive occupational medical actions.

## Methods

### Study population

**Fig. 1:**
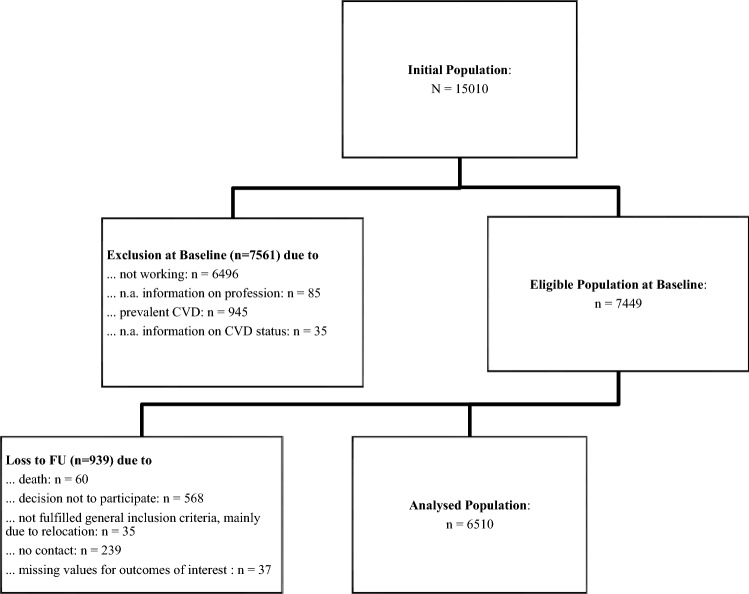
Flow chart study population

Subjects were participants of the GHS, a population-based, prospective cohort study conducted in the city of Mainz and the adjacent district of Mainz-Bingen in Rhineland-Palatinate (RLP), Germany. Baseline examinations were carried out between 2007 and 2012 with follow-up examinations five years later. Details of the study design have been published elsewhere (Wild et al. 2012). For the present analyses, only working participants without prevalent CVD at baseline who finished the follow-up and had no missing values for outcomes of interest were eligible (*n* = 6510) (Fig. 1). The exclusion of participants with prevalent CVD at baseline should also reduce the risk of reverse causation due to changed health behavior after a CVD diagnosis.

### Assessment of occupational and other socio-demographic characteristics

The assignment of occupational groups was done using the occupational status at baseline categorized according to the official German occupational classification system “KldB 2010” (German Federal Employment Agency 2020; Prigge et al. 2014). Self-reported information on occupational history with up to 15 previous occupational periods was available. We divided the group of professionals from the HSE fields (KldB job category 8) into either teachers (*n* = 215) or *other* HSE professionals (“HSE_OTHERS”: *n* = 1061). The teachers group included teachers and headmasters at primary, secondary, special (for children with disabilities or learning difficulties) and vocational schools or training colleges (Table 5 in “Appendix”). “HSE_OTHERS” comprised other educational and social occupations, medical and non-medical health professions, domestic sciences and theologians (Table 5 in “Appendix”).The occupational group “non-HSE” (*n* = 5234) included all other GHS participants working in other fields.

All analyses were stratified by sex. Regarding the regression analyses, we first adjusted for age (adjustment model 1). Furthermore we controlled for the duration in the occupational group to avoid possible bias due to systematic differences between the occupational groups (adjustment model 2). For that we assessed the years of belonging to the occupational group until baseline.

We also considered socio-economic status (SES) (adjustment model 3). SES-score allocation was based on school education, professional education, occupational position and salary (Lampert et al. 2013). SES-scores ranged from 3 to 21 with higher scores indicating a higher SES. The group-specific variance of SES differs depending on the composition of the occupational group, so by comparing occupational groups SES is partly taken into consideration. However, we were also interested in the SES-independent effects of teaching on cardiovascular health.

### Outcome measures

#### CVD risk factors and CVD

We examined the prevalence of behavior-related and physiological CVD risk factors at baseline. Smoking was defined as self-reported active smoking (including less than one cigarette per day) within the last 6 months. Information on alcohol intake was based on self-reported amounts. We considered the unhealthy intake above the recommended limit to be an alcohol intake > 10 g/day for women and an intake > 20 g/day for men (Deutsche Gesellschaft fuer Ernaehrung et al. 2000). Physical inactivity, or here: “no active sport”, was assessed based on responses to the SQUASH physical activity questionnaire (Wendel-Vos 2003). There, among other information on physical activity, participants were asked to report frequencies, durations and intensities for up to five sport activities. “Physical inactivity” means that the participant failed to take part in any sport activity with a minimum intensity of two metabolic equivalents for task (MET) even once a week.

Obesity was defined as a BMI ≥ 30 kg/m^2^, measured as weight in kg divided by height in m^2^ (WHO 2000). Hypertension was defined as an intake of antihypertensive drugs, a mean systolic blood pressure ≥ 140 mm Hg or a mean diastolic blood pressure ≥ 90 mm Hg in three consecutive measurements at rest or a self-reported doctor-diagnosed arterial hypertension. Diabetes was defined as the condition of HbA1c-level of ≥ 6.5%, an intake of anti-diabetic medication or self-reported doctor-diagnosed diabetes. We defined dyslipidemia as LDL/HDL-ratio of ≥ 3.5, triglycerides > 150 mg/dl, the intake of lipid modifying medication (ATC code C10) or its self-reported physician diagnosis. Family history of myocardial infarction (MI) or stroke was defined as at least one MI or stroke in a female first-degree relative before 65 years of age or in a male first-degree relative before 60 years of age.

Furthermore, we estimated the proportion of incident self-reported doctor-diagnosed CVD per occupational group. For that purpose we assessed CVD by means of the answers to the question *“Have you been diagnosed by a physician with coronary artery disease/myocardial infarction/stroke/atrial fibrillation/congestive heart failure/peripheral arterial disease since baseline?”* We considered at least one self-reported condition as a new case of CVD.

### Statistical analysis

All of the statistical analyses were stratified by sex. To describe the study population, we present the quantitative variables “age”, “SES” and “duration in the occupational group” with mean values and standard deviations. For each CVD risk factor, we report the prevalence (proportion) at baseline per occupational group. To compare teachers with the other occupational groups (HSE_OTHERS and non-HSE), we defined teachers as the reference group and estimated the prevalence ratio (PR) for each CVD risk factor at baseline using robust Poisson regression analysis. The regression models were incrementally adjusted for age (model 1), the duration in the occupational group (model 2) and SES (model 3). We also calculated the sex-stratified crude CVD incidence rate in each occupational group based on the 5-year cumulative incidence of self-reported doctor-diagnosed CVD at follow-up. We also estimated age-weighted incidence proportions. For standardization we used the age-structure of the “German Working Population” (31.12.2018, Federal Statistics Office 2020) and weighted per 5-year age groups. We report the 95% confidence interval for the estimates of regression analyses and for weighted incidence proportions. Due to the exploratory character of our study, we did not correct for multiple testing. Statistical analyses were performed using the statistical software R, version 3.6.0 (2019-04-26).

## Results

### Characteristics of the study population

Teachers had the highest average age compared to the other occupational groups, with an average age of 53.2 years in male and 49.5 years in female teachers (Table 1). Teachers also had a higher SES and had been longer in their job until baseline than members of the other occupational groups (Table 1).

### Prevalence of CVD risk factors

Results of regression analyses on prevalent CVD risk factors are shown in Tables 2, 3. In the only age-adjusted model, we observed a twofold increased prevalence of smoking in male non-HSE workers compared to male teachers (PR 2.26; 95%-CI: 1.06–4.82). The difference was less pronounced in women (PR 1.44, 95%-CI: 0.98–2.11). Male non-HSE workers were also more likely to be physically inactive than male teachers (PR 1.89; 95%-CI: 1.24–2.87). Corresponding differences were not seen in women. In contrast, male and female other HSE professionals, as well as non-HSE workers, reported alcohol intakes above the recommended limit less frequently than teachers (e.g., in females: HSE_OTHERS vs. teachers: PR 0.66; 95%-CI 0.51–0.84 and non-HSE vs. teachers: PR 0.71; 95%-CI: 0.57–0.89). Except for family history of myocardial infarction or stroke in women (female non-HSE vs. teachers PR 1.54; 95%-CI: 1.06–2.23) there were no remarkable differences between teachers and other HSE professionals or non-HSE workers in the prevalence of the other risk factors (i.e., obesity, hypertension, diabetes or dyslipidemia).

**Table 1:** Baseline demographic data on teachers, other professionals of the health, social or educational fields (HSE_OTHERS) and non-HSE workers, stratified by sex

Men	HSE_OTHERS (*n* = 309)	Non-HSE (*n* = 3172)	Teachers (*n* = 63)
	Mean (Standard deviation)	Mean (Standard deviation)	Mean (Standard deviation)
Age (years)	49.6 (8.4)	48.6 (8.1)	53.2 (8.3)
SES (score)	17.5 (3.3)	14.5 (4.3)	19.4 (1.7)
Duration in the occupational group (years)	14.6 (10.1)	14.9 (10.7)	18.6 (11.7)

Women	HSE_OTHERS (*n* = 752)	Non-HSE (*n* = 2062)	Teachers (*n* = 152)
	Mean (Standard deviation)	Mean (Standard deviation)	Mean (Standard deviation)
Age (years)	48.5 (7.4)	48.1 (7.5)	49.5 (8.3)
SES (score)	14.3 (3.6)	13.1 (3.9)	18.8 (2.1)
Duration in the occupational group (years)	13.2 (10.4)	13.1 (10.1)	14.8 (11.3)

**Table 2:** In **men**: Prevalence of CVD risk factors at baseline (columns 2–4) and Prevalence Ratio (PR) (columns 6–7) by occupational group (reference group: teachers), with basic and further adjustment (Models 1–3)*; based on robust generalized log-linear Poisson regression models

Risk factor	Prevalence (proportion) of CVD risk factors at baseline in men by occupational group			Adjustment	PR (95%-CI)	
	HSE_OTHERS (*n* = 309)	Non-HSE (*n* = 3172)	Teachers (*n* = 63)		HSE_OTHERS vs. teachers	Non-HSE vs. teachers
Smoking (yes)	15.5% (48/309)	23.3% (738/3170)	**9.5% (6/63)**	M1	1.53 (0.69–3.40)	**2.26 (1.06–4.82)**
				M2	1.52 (0.68–3.37)	**2.25 (1.06–4.80)**
				M3	1.30 (0.59–2.88)	1.58 (0.74–3.38)
Unhealthy alcohol intake (yes)	26.9% (83/309)	28.6% (905/3168)	**42.9% (27/63)**	M1	**0.69 (0.49–0.96)**	0.76 (0.57–1.01)
				M2	**0.69 (0.50–0.97)**	0.76 (0.57–1.01)
				M3	0.74 (0.53–1.03)	0.90 (0.67–1.21)
Physical inactivity (no sport) (yes)	37.2% (115/309)	45.5% (1443/3172)	**25.4% (16/63)**	M1	1.53 (0.98–2.38)	**1.89 (1.24–2.87)**
				M2	1.54 (0.99–2.39)	**1.90 (1.25–2.88)**
				M3	1.34 (0.86–2.09)	1.36 (0.89–2.09)
Obesity (yes)	13.3% (41/309)	23.1% (732/3172)	**15.9% (10/63)**	M1	0.88 (0.47–1.66)	1.56 (0.88–2.75)
				M2	0.88 (0.47–1.65)	1.55 (0.88–2.75)
				M3	0.77 (0.41–1.45)	1.13 (0.63–2.02)
Hypertension (yes)	35.0% (108/309)	43.0% (1363/3172)	**49.2% (31/63)**	M1	0.82 (0.61–1.08)	1.05 (0.82–1.34)
				M2	0.83 (0.62–1.09)	1.06 (0.83–1.35)
				M3	0.81 (0.61–1.07)	1.00 (0.78–1.28)
Diabetes (yes)	6.1% (19/309)	5.3% (168/3170)	**6.3% (4/63)**	M1	1.21 (0.43–3.36)	1.12 (0.44–2.88)
				M2	1.20 (0.43–3.34)	1.12 (0.44–2.87)
				M3	0.99 (0.35–2.76)	0.69 (0.26–1.82)
Dyslipidemia (yes)	41.7% (129/309)	47.2% (1497/3170)	**42.9% (27/63)**	M1	1.02 (0.75–1.40)	1.17 (0.88–1.56)
				M2	1.02 (0.75–1.40)	1.17 (0.88–1.56)
				M3	0.99 (0.73–1.36)	1.09 (0.82–1.46)
Family history of MI or stroke (yes)	22.3% (69/309)	19.9% (632/3172)	**17.5% (11/63)**	M1	1.31 (0.73–2.34)	1.18 (0.68–2.03)
				M2	1.33 (0.75–2.36)	1.19 (0.69–2.04)
				M3	1.28 (0.72–2.27)	1.07 (0.62–1.86)

^*^Model 1 (M1): adjusted for age; Model 2 (M2): adjusted for age and duration in the occupational group; Model 3 (M3): adjusted for age, duration in the occupational group and SES

**Table 3:** In **women**: Prevalence of CVD risk factors at baseline (columns 2–4) and Prevalence Ratio (PR) (columns 6–7) by occupational group (reference group: “teachers”), with basic and further adjustment (Models 1–3)*; based on robust generalized log-linear Poisson regression models

Risk factor	Prevalence (proportion) of CVD risk factors at baseline in women by occupational group			Adjustment	PR (95%-CI)	
	HSE_OTHERS (*n* = 752)	Non-HSE (*n* = 2062)	Teachers (*n* = 152)		HSE_OTHERS vs. teachers	Non-HSE vs. teachers
Smoking (yes)	20.1% (151/752)	22.0% (454/2062)	**15.1% (23/152)**	M1	1.32 (0.88–1.97)	1.44 (0.98–2.11)
				M2	1.31 (0.88–1.97)	1.43 (0.97–2.11)
				M3	0.93 (0.62–1.41)	0.94 (0.63–1.40)
Unhealthy alcohol intake (yes)	22.9% (172/752)	24.7% (509/2062)	**35.5% (54/152)**	M1	**0.66 (0.51–0.84)**	**0.71 (0.57–0.89)**
				M2	**0.66 (0.51–0.84)**	**0.72 (0.57–0.90)**
				M3	0.87 (0.68–1.13)	1.03 (0.81–1.30)
Physical inactivity (no sport) (yes)	36.2% (272/752)	38.8% (801/2062)	**37.5% (57/152)**	M1	0.98 (0.78–1.23)	1.06 (0.86–1.31)
				M2	0.98 (0.79–1.23)	1.06 (0.86–1.31)
				M3	**0.69 (0.54–0.87)**	**0.68 (0.54–0.85)**
Obesity (yes)	14.9% (112/751)	19.1% (393/2061)	**15.1% (23/152)**	M1	1.01 (0.66–1.53)	1.30 (0.88–1.92)
				M2	1.01 (0.66–1.53)	1.30 (0.88–1.92)
				M3	0.66 (0.43–1.01)	0.76 (0.50–1.15)
Hypertension (yes)	28.1% (211/751)	30.8% (634/2061)	**27.0% (41/152)**	M1	1.13 (0.86–1.49)	1.27 (0.98–1.64)
				M2	1.14 (0.87–1.49)	1.27 (0.99–1.65)
				M3	0.98 (0.74–1.30)	1.05 (0.80–1.38)
Diabetes (yes)	2.4% (18/748)	3.0% (61/2053)	**2.6% (4/151)**	M1	0.99 (0.34–2.86)	1.24 (0.46–3.36)
				M2	0.98 (0.34–2.84)	1.23 (0.45–3.34)
				M3	0.70 (0.24–2.04)	0.80 (0.28–2.30)
Dyslipidemia (yes)	23.8% (179/751)	21.0% (433/2059)	**21.9% (33/151)**	M1	1.15 (0.83–1.60)	1.03 (0.75–1.41)
				M2	1.16 (0.83–1.61)	1.03 (0.75–1.42)
				M3	0.87 (0.61–1.22)	0.71 (0.51–1.00)
Family history of MI or stroke (yes)	22.3% (168/752)	23.5% (485/2062)	**15.8% (24/152)**	M1	1.45 (0.98–2.13)	**1.54 (1.06–2.23)**
				M2	1.45 (0.99–2.14)	**1.54 (1.06–2.24)**
				M3	1.32 (0.89–1.97)	1.37 (0.93–2.01)

^*^Model 1 (M1): adjusted for age; Model 2 (M2): adjusted for age and duration in the occupational group; Model 3 (M3): adjusted for age, duration in the occupational group and SES

The adjustment for the duration in the occupational group had no relevant impact on the results. In contrast, adjustment for SES generally reduced the observed effects towards the NULL. One exception was observed for physical inactivity in females. In the additionally SES-adjusted model, female other HSE professionals as well as female non-HSE workers showed a substantial lower prevalence of physical inactivity than female teachers (in females: HSE_OTHERS vs. teachers: PR 0.69; 95%-CI: 0.54–0.87 and non-HSE vs. teachers: PR 0.68; 95%-CI: 0.54–0.85).

### Incidence of CVD

Based on the 5-year incidence of self-reported doctor-diagnosed CVD at follow-up (Table 4), the crude incidence rate was 31.7 cases per 10,000 person-years among male teachers and 13.2 cases per 10,000 person-years in female teachers. Among other HSE professionals there were 45.3 cases per 10,000 person-years in men and 16.0 cases per 10,000 person-years in women. The crude incidence rate for non-HSE workers was 80.1 cases per 10,000 person-years in men and 40.7 cases per 10,000 person-years in women. No substantial difference in age-weighted cumulative incidence was detected between teachers and other HSE professionals or non-HSE workers (Table 4).

**Table 4:** Observed and age-weighted* cumulative incidence of self-reported doctor-diagnosed CVD at 5-year follow-up, stratified by sex

Men	Observed 5-year cumulative incidence of CVD in % (n/N) and crude incidence rate per 10,000 person-years	Age-weighted* 5-year cumulative incidence of CVD with (95%-CI)
HSE_OTHERS	2.27% (7/309); 45.3 cases per 10,000 person-years	3.23% (1.65%–6.02%)
Non-HSE	4.00% (127/3172); 80.1 cases per 10,000 person-years	4.32% (3.64%–5.11%)
Teachers	1.59% (1/63); 31.7 cases per 10,000 person-years	2.10% (0.24%–9.81%)

Women	Observed 5-year cumulative incidence of CVD in % (n/N) and crude incidence rate per 10,000 person-years	Age-weighted 5-year cumulative incidence of CVD with (95%-CI)
HSE_OTHERS	0.80% (6/752); 16.0 cases per 10,000 person-years	1.37% (0.72%–2.55%)
Non-HSE	2.04% (42/2062); 40.7 cases per 10,000 person-years	2.42% (1.83%–3.19%)
Teachers	0.66% (1/152); 13.2 cases per 10,000 person-years	1.12% (0.18%–4.59%)

^*^Standard population: “German Working Population”(31.12.2018, Federal Statistics Office 2020), weighted per 5-year age groups and sex

## Discussion

Using data from the GHS cohort, we examined the prevalence of especially behavioral and physiological CVD risk factors at baseline as well as the cumulative incidence of self-reported doctor-diagnosed CVD at follow-up in teachers, other HSE professionals and non-HSE workers from Rhineland-Palatinate, Germany. Results from the occupational comparisons indicate potentially heightened risks for teachers that could guide the development of effective preventative occupational health and safety measures.

Due to our exploratory approach, we did not control for multiple testing. Therefore, the reported results should be viewed with some caution. Nevertheless, our results indicate differences between teachers and particularly non-HSE workers with regard to the prevalence of some CVD risk factors at baseline. Considering physical inactivity, the prevalence of inactivity among teachers was only about half of that reported by male non-HSE workers. Furthermore, smoking prevalence was particularly low among male teachers in contrast to male non-HSE workers. These results correspond to previously described results (e.g., Seibt et al. 2016; Scheuch et al. 2015; Gilbert et al. 2015). However, unlike the results on the smoking prevalence among French teachers reported by Gilbert et al (2015), in our study occupational group-related differences in smoking attenuated after SES-adjustment.

Teachers in our study were more likely to report an alcohol intake above the recommended limit compared to other professionals of the HSE fields or non-HSE workers. Other studies (e.g., RKI (Hrsg) 2014) have found increased alcohol intake is associated with higher SES, and this association is usually more pronounced in women. With regard to differences in alcohol intake between teachers and other occupations, study results are not consistent. Kovess-Masféty et al. (2006) did not report significant differences in alcohol abuse or dependency between teachers and control persons in France. In the study by Seibt et al. (2016), male and female teachers were significantly less likely to report consuming alcohol regularly than participants of the regional working sample. At the same time, the percentage of those who reported drinking *no* alcohol was two to three times higher in the regional sample compared to the teachers group. However, it is unclear, the extent to which ex-drinkers who gave up consuming alcohol belong to this group. Gilbert et al. (2015) found healthy behavior in different aspects more pronounced among teachers than among other occupations, but could not confirm this finding regarding alcohol consumption.

Some studies reported a heightened risk of hypertension for teachers compared to the general population (Scheuch et al. 2015; Seibt et al. 2016). Others reported a lower risk of hypertension for (female) teachers compared to office workers (Seibt et al. 2005) and nurses (Brown et al. 2006), respectively. In our study, the teachers did not differ remarkably from the other HSE professionals or non-HSE workers with regard to hypertension. Although there is some evidence against the assumption that job strain increases resting blood pressure (Nyberg et al. 2013), previous results are not at all consistent (Gilbert-Ouimet et al. 2014). Lastly, regardless of occupational comparisons, the hypertension prevalence of 49.2% (Table 2) in male teachers indicates a potential area for improvement in the context of occupational health care.

Finally, we estimated the age-weighted 5-year cumulative incidence of self-reported CVD to be 2.10%; 95%-CI: 0.24–9.81% for male teachers and 1.12%; 95%-CI: 0.18–4.59% for female teachers (Table 4). However, CVD events were rare and resulted in wide confidence intervals. So the incidence results should be interpreted with caution, and this imprecision makes it difficult to detect differences between the groups of teachers and other HSE professionals or non-HSE workers. Helmert et al. (1997) analyzed data from population health surveys from 1984–1991 in Western Germany, and found that of the 30 most common occupations among men, teachers had the lowest CVD prevalence. However, only few mainly blue-collar occupations among men, and only kindergarten teachers and cooks among women had a CVD prevalence significantly higher than that of teachers (Helmert et al. 1997). Seibt et al. (2005), on the other hand, surveyed risk factors and resources of work ability in female secondary school teachers and female office workers, and detected no significant differences between those two occupational groups regarding CVD prevalence. Overall, the choice of the comparison group(s) may make a substantial difference.

### Strengths and limitations

Participants with prevalent CVD at baseline were excluded to prevent reverse causation (e.g., change of occupational group due to CVD diagnosis). Furthermore, this should reduce any possible healthy worker bias specific to certain occupational groups. High job demands on the one hand, and civil servant status on the other hand might make teachers more likely to retire early after a CVD diagnosis than workers in less demanding jobs and without a civil servant status. A further strength of our study was that we could evaluate teachers’ cardiovascular health in contrast to other working groups of the GHS population without a needing to obtain external data or use surveys for comparisons.

One limitation of our study was the lack of study power. Besides the low number of incident cases, our analyses were based on self-reported doctor-diagnosed outcomes, which might affect data validity. Nevertheless, data validity varies depending on the diagnoses considered (Machón et al. 2013; Bergmann et al. 1998). Moreover, using a combined CVD outcome, as we did, may compensate for a single misclassified self-reported cardiovascular diagnosis (Bergmann et al. 1998). A further limitation may result from not considering people who died during the five year FU to calculate incidences. This may have led to a reduction of incident cardiac events, but there is no reason to assume that the distribution of fatal cardiac events would differ from that of non-fatal CVD among the occupational groups.

Data on the cardiovascular health of teachers is rare, so the analysis of teachers in the GHS cohort was seen as a unique possibility to assess cardiovascular health in a sample of teachers in RLP. The proportion of women in the analyzed group of GHS teachers corresponds to the proportion of women in the general population of teachers in RLP (GHS: 70.7% vs. RLP: 71.2%). However, the average age of the group of GHS teachers (50.6 years) exceeds the average age of the general population of teachers in RLP (44.7 years) (Letzel et al. 2019). This is probably due to the fact that study participants had to be aged 35 years or older at baseline. This discrepancy has to be taken into account particularly if absolute results are generalized to entire population of teachers in RLP. Furthermore, teachers’ work circumstances may vary between school types, regions and countries. This also limits the possibilities of extensive generalization of our study results. Moreover, the study is based on extensive interviews that require a certain level of health, language skills, interest, and willingness to participate (Daubenbuechel 2014). However, while this limits the external validity, it should affectless the (internal) group comparisons.

## Conclusion

Like previous study results, we found no substantial indications of a heightened CVD risk among teachers. With a focus on the CVD risk factors of teachers as a well-defined homogenous occupational group, our study results suggest potential areas for preventive measures in the occupational health care of teachers. While our results indicate a basically healthier lifestyle of male teachers, especially regarding the avoidance of the CVD risk factors, such as physical inactivity and smoking. Our results also indicate a relatively heightened prevalence of unhealthy alcohol intake in female and male teachers, and a high prevalence of hypertension in male teachers (albeit comparable to the high prevalence of hypertension in other occupational groups). In this respect, our results should be considered during routine occupational-medical care and research on teachers’ health.

## Appendix

See Table 5.

**Table 5:** Definition of the occupational groups “teachers” and “HSE_OTHERS” in GHS according to KldB2010 with number of participants

Group	Category 8 (KldB 2010)	Example	*n*
Teachers	+ Teaching profession	Teachers at secondary schools, teachers at primary schools, headmasters	215
HSE_OTHERS	+ Medical health professions (no. 81)	Physicians, nurses, psychologists	508
	+ Non-medical health professions (no. 82)	Geriatric nurses, wellness and cosmetic professions	136
	+ Educational and social occupations, domestic sciences, theology (no. 83)	Kindergarten teachers, social workers, theologians	249
	+ Rest of (no. 84)	Educators and teachers at extracurricular institutions, university professors	168
