# Correction to: Self‑reported cardiovascular health of teachers: results from the 5‑year follow‑up of the Gutenberg Health Study cohort

**DOI:** 10.1007/s00420-021-01747-2

**Published:** 2021-07-30

**Authors:** Merle Riechmann‑Wolf, Sylvia Jankowiak, Andreas Schulz, Janice Hegewald, Karla Romero Starke, Falk Liebers, Karin Rossnagel, Alicia Poplawski, Natalie Arnold, Matthias Nübling, Andreas Seidler, Manfred Beutel, Norbert Pfeiffer, Karl Lackner, Thomas Münzel, Kathrin Bogner, Philipp S. Wild, Ute Latza, Stephan Letzel

**Affiliations:** 1 Institute for Teachers’ Health, University Medical Center of the Johannes Gutenberg University of Mainz, Kupferbergterrasse 17-19, 55116 Mainz, Germany; 2 Federal Institute for Occupational Safety and Health, BAuA Berlin, Berlin, Germany; 3 University Medical Center of the Johannes Gutenberg University of Mainz, Mainz, Germany; 4 IPAS Dresden: Institute and Polyclinic of Occupational and Social Medicine, Carl Gustav Carus Faculty of Medicine, TU Dresden, Dresden, Germany; 5 FFAW: The Freiburg Research Center for Occupational Sciences, Freiburg, Germany; 6 Institute of Occupational, Social, and Environmental Medicine, University Medical Center of the Johannes Gutenberg University of Mainz, Mainz, Germany

## Correction to: International Archives of Occupational and Environmental Health (2021) 94:251–259 10.1007/s00420-020-01576-9

The article: Self‑reported cardiovascular health of teachers: results from the 5‑year follow‑up of the Gutenberg Health Study cohort, written by Merle Riechmann‑Wolf· Sylvia Jankowiak2 · Andreas Schulz3 · Janice Hegewald· Karla Romero Starke ·Falk Liebers2 · Karin Rossnagel· Alicia Poplawski · Natalie Arnold· Matthias Nübling· Andreas Seidler ·Manfred Beutel· Norbert Pfeiffer · Karl Lackner · Thomas Münzel · Kathrin Bogner · Philipp S. Wild · Ute Latza ·Stephan Letzel, was originally published electronically on the publisher’s internet portal (currently SpringerLink) on 26 October 2021 without open access.

With the author(s)’ decision to opt for Open Choice, the copyright of the article changed on 15 August 2021 © The Author(s) 2021 and the article is forthwith distributed under the terms of the Creative Commons Attribution 4.0 International License (http://creativecommons.org/licenses/by/4.0/), which permits use, duplication, adaptation, distribution and reproduction in any medium or format, as long as you give appropriate credit to the original author(s) and the source, provide a link to the Creative Commons license and indicate if changes were made.

**Open Access** This article is distributed under the terms of the Creative Commons Attribution 4.0 International License (http://creativecommons.org/licenses/by/4.0/), which permits unrestricted use, distribution, and reproduction in any medium, provided you give appropriate credit to the original author(s) and the source, provide a link to the Creative Commons license, and indicate if changes were made.

The original article was corrected.

